# L1-Constrained Fractional-Order Gradient Descent for Axial Dimension Estimation of Conical Targets

**DOI:** 10.3390/s25165082

**Published:** 2025-08-15

**Authors:** Yue Dai, Shiyuan Zhang, Guoqiang Guo

**Affiliations:** Nanjing Research Institute of Electronic Technology, Nanjing 210039, China; daiyue20018@163.com (Y.D.); guoguoq3303@163.com (G.G.)

**Keywords:** High-Range Resolution Profiles (HRRPs), fractional-order gradient descent, L1 norm, size estimation

## Abstract

The efficient utilization of structural information in High-Range Resolution Profiles (HRRPs) is of great significance for improving recognition performance. This paper proposes a size estimation method based on L1-norm variable fractional-order gradient descent, which achieves size inversion in complex electromagnetic environments by establishing an HRRP projection model of ballistic targets. Specifically: First, through rigorous geometrical optics analysis, an analytical relationship model between the target’s projected size and actual size is established. Second, an error function under the L1-norm is constructed, and an adaptive order-adjusting fractional-order gradient descent method is employed for optimization, effectively overcoming the sensitivity to outliers inherent in traditional L2-norm methods. Finally, by introducing a dynamic order-switching mechanism, computational efficiency is improved while ensuring convergence accuracy. Experimental results show that at a measurement error of 0.4 m, the proposed method maintains excellent estimation performance with sensitivity to outliers reduced, and the actual size inversion error remains stable below 3.7%.

## 1. Introduction

In complex electromagnetic environments, target recognition [1,2] and parameter estimation [3] remain pivotal challenges in radar signal processing. High-Range Resolution Profiles (HRRPs) [4,5], which effectively characterize the scattering properties of targets, are particularly valuable for non-cooperative target identification due to their unique advantages—enabling the acquisition of fine structural features without motion compensation [6,7]. Nevertheless, existing approaches for estimating target parameters using one-dimensional range profiles still suffer from significant limitations.

Traditional approaches to axial dimension estimation—that is, determining the length of a target along its principal axis—can be categorized into three main types: (1) Maximum Likelihood Estimation (MLE)-based optimization methods, such as the precession model parameter estimation proposed in [8], construct likelihood functions involving five parameters (e.g., size; precession frequency). While theoretically sound, these methods exhibit inherent limitations, including susceptibility to local optima in multi-parameter optimization, high computational complexity, and stringent requirements on data sampling rates [9]. (2) Parametric fitting methods, which rely on strong assumptions (e.g., fixed attitude angles), have limited practical applicability [10]. (3) Conventional L2-norm-based least squares estimation techniques [11] are overly sensitive to outliers [12], leading to degraded robustness in complex electromagnetic environments.

On the other hand, classical integer-order calculus exhibits inherent limitations when addressing complex systems with nonlocality and memory effects [13]. Fractional-order calculus, a natural extension of integer-order calculus, introduces non-integer differential operators to better describe physical processes with memory and hereditary properties [14,15,16]. This method has been applied in various disciplines, including physics, biology, and engineering. Notably, fractional-order gradient descent algorithms achieve a flexible trade-off between convergence speed and precision by adjusting the differential order (α). This adaptability effectively mitigates the long-standing conflict between convergence speed and accuracy in conventional optimization methods [17,18].

To overcome these challenges, this study introduces fractional-order optimization theory into axial dimension estimation and proposes an adaptive hybrid-order fractional gradient descent (OFGD) algorithm. By replacing the conventional precession model assumption with a direct linear mapping relationship for one-dimensional range profile projection, the parameter space is reduced from five dimensions to one. Compared to existing methods, the key contributions of this work include the following: (1) Intelligent Order Adaptation Mechanism: A dynamic order-switching strategy is proposed, where a high initial order (α > 1) ensures rapid convergence, while a monotonically decreasing function adjusts the order when the gradient magnitude falls below a threshold. Theoretical analysis proves that this approach reduces iteration counts while maintaining low steady-state error. (2) Enhanced Anti-Interference Capability: An L1-norm-constrained [19] robust optimization model significantly mitigates outlier effects. Additionally, the algorithm’s computational complexity is reduced to O(n), with experimental data confirming decreased iteration time.

## 2. Target Motion Model

### 2.1. Geometric Model of Scattering Centers

**Figure 1:**
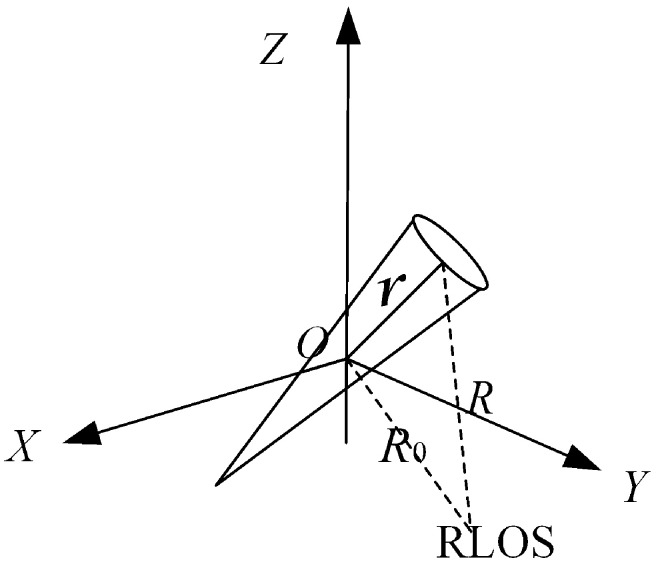
Schematic diagram of the target scattering imaging signal model.

In the high-frequency scattering regime, the scattering from man-made targets exhibits “sparsity”. This means that the overall target scattering is not contributed by the entire target surface, but can be fully characterized by a series of discrete scattering centers. Consequently, the total target scattering echo received by the radar can be modeled as the vector superposition of echoes from these discretely distributed, independent scatterers. As illustrated in the geometry schematic for radar observation of a target in Figure 1, the radar echo from an extended target can then be expressed as follows:

(1) $$S_{r}\left(k\right)=K_{0}\exp\left(j2k\;\cdot \;R_{0}\right)\;\cdot \;S_{t}\left(k\right)\;\cdot \;\underset{n^{3}}{\;\int \int \int \;}\Gamma \left(r,k\right)\exp\left(-j2k\;\cdot \;r\right)dr$$

where k is the spatial wavenumber vector, with magnitude 2πf/c and direction aligned along the radar line-of-sight toward the target center; $R_{0}$ is the distance vector from the target’s phase reference center to the radar; r is the position vector of local scattering centers relative to the target reference center; $K_{0}$ is a constant factor related to antenna gain and propagation attenuation; $S_{t}(k$) is the radar transmitted waveform; $D^{3}$ is the spatial volume enclosing the three-dimensional extended target; Γ(r,k) is the target scattering distribution function. If the radar transmitted signal has a uniform spatial spectral distribution over the entire observation wavenumber domain, then

(2) $$|S_{t}\left(k\right)|=S_{0},\;k\in K_{e}$$

At this point, Equation (1) can be simplified as

(3) $$S_{t}\left(k\right)=G_{0}\;\cdot \;\underset{D^{3}}{\;\int \int \int \;}\Gamma \left(r,k\right)\exp\left(-j2k\;\cdot \;r\right)dr$$

where $G_{0}$ is a complex constant that can be neglected, yielding

(4) $$S_{t}\left(k\right)=\underset{D^{3}}{\;\int \int \int \;}\Gamma \left(r,k\right)\exp\left(-j2k\;\cdot \;r\right)dr$$

According to the Geometrical Theory of Diffraction (GTD) [20], the radar echo represented by Equation (4) can be rewritten as

(5) $$S_{m}\left(f,\theta \right)=\sum_{m=1}^{M}A_{m}{\left(\frac{f}{f_{c}}\right)}^{\alpha_{m}}\exp\left(-j\frac{4\pi fr_{m}}{c}\right)$$

where *v* denotes the polarization combination; θ is the azimuth angle of the radar line-of-sight relative to the target axis; *M* represents the number of scattering centers; $A_{m}$ is the complex scattering amplitude of the *m*-th scattering center; ${\left(f/f_{c}\right)}^{\alpha_{m}}$ describes the frequency-dependent behavior of the scattering intensity for this scattering center, where $\alpha_{m}$ represents the geometric type of the scattering center, taking values as integer multiples of 0.5, which reflects the geometric characteristics of the scattering center but is independent of polarization; $r_{m}$ is the projection distance of r along the radar line-of-sight direction.

Assuming the radar transmits a wideband linear-frequency-modulated (LFM) signal with carrier frequency $f_{c}$, chirp rate μ, and sampling period $T_{s}$, the radar signal frequency corresponding to the *n*-th sampling instant $t_{n}=nT_{s}+t_{n0}$ within a pulse repetition interval is given by

(6) $$f_{n}=f_{c}+\mu \;\cdot \;t_{n}=f_{c}+\mu \left(nT_{s}+t_{n0}\right)$$

where *n* takes values −N/2+1,−N/2+2,…,N/2 (*N* is an even number representing the effective number of samples); $t_{n0}$ denotes the sampling start time.

Setting $t_{n0}=0$, the one-dimensional scattering image echo of the target at a fixed azimuth angle is expressed as

(7) $$S_{n}\left(n\right)=\sum_{m=1}^{M}a_{m}{\left(1+n\frac{\mu T_{s}}{f_{c}}\right)}^{\alpha_{m}}\exp\left(jn\omega_{m}\right)$$

where

(8) $$a_{m}=A_{m}\exp\left(-j\frac{4\pi f_{c}r_{m}}{c}\right)$$

(9) $$\omega_{m}=-j\frac{4\pi \mu T_{s}r_{m}}{c}$$

By approximating the exponential function, the one-dimensional scattering imaging signal model of the target can be expressed as

(10) $$S_{n}\left(n\right)=\sum_{m=1}^{M}a_{m}e^{(-d_{m}+j\omega_{m})n}=\sum_{m=1}^{M}a_{m}P_{m}^{n}$$

where *M* is the model order, representing the number of equivalent scattering centers; $a_{m}$ is the complex amplitude coefficient of the model, characterizing the scattering strength of the scattering center; $P_{m}=e^{-d_{m}+j\omega_{m}}$ is the model pole, where $d_{m}$ is the attenuation factor describing the geometric type of the scattering center, and $\omega_{m}$ is the model resonant frequency. The model parameters ${\left\{a_{m},d_{m},\omega_{m}\right\}}_{m=1}^{M}$ completely characterize the scattering strength, geometric types, and radial distance distribution of the multiple scattering centers of the target.

### 2.2. Trajectory Reconstruction

To simplify the analysis, the following fundamental assumptions are adopted:

- Atmospheric effects are ignored during target flight, and perturbation factors including Earth’s oblateness and gravitational forces from celestial bodies are disregarded;
- The radar is assumed to be capable of detecting all targets within its observation range.

The state description during the midcourse flight phase of the target corresponds to a two-body problem; hence the North-Sky-East (NSE) coordination and the geocentric inertial coordination are employed. Define the North-Sky-East (NSE) coordination S−rθδ, where the origin *S* coincides with the target point, the *r*-axis passes through the geocenter toward the zenith, the θ-axis is perpendicular to the target’s meridian plane pointing due east, and the δ-axis is determined by the right-hand rule pointing due north. This coordination is time-varying.

**Figure 2:**
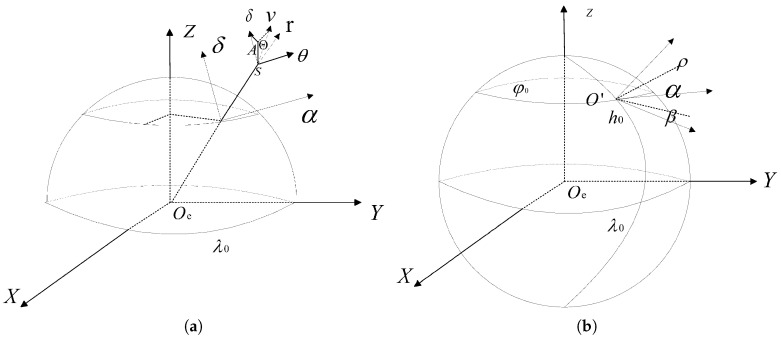
(**a**) The relationship between the North-Sky-East (NSE) and the geocentric inertial coordination. (**b**) The transformation relationship between radar measurement and the geocentric inertial coordination.

Define the geocentric inertial coordination $O_{e}-XYZ$, where the origin $O_{e}$ coincides with the Earth’s center, the *X*-axis lies in the equatorial plane passing through the vernal equinox, the *Y*-axis lies in the equatorial plane perpendicular to the *X*-axis, and the *Z*-axis is determined by the right-hand rule pointing toward the North Pole. The relationship between the North-Sky-East (NSE) coordination and the geocentric inertial coordination is illustrated in Figure 2a. In $O_{e}-XYZ$, the target’s position and velocity vectors are as follows: $r={\left[x\;y\;z\right]}^{T}$, $v={\left[\dot{x}\;\dot{y}\;\dot{z}\right]}^{T}$. In S−rθδ, the position and velocity vectors are as follows: $r_{s}\;=\;{\left[0\;0\;0\right]}^{T}$, $v_{s}={\left[\dot{r}\;r\cos\delta \;\cdot \;\dot{\theta }\;r\dot{\delta }\right]}^{T}$.

Define the flight azimuth angle *A* as the angle between the projection of velocity vector v on the Sθδ plane and the δ-axis, measured clockwise from the δ-axis: 0≤A≤2π. Define the velocity pitch angle Θ as the angle between the velocity vector v and the Sθδ plane, positive when above the local horizontal plane: $-\frac{\pi }{2}\leq \Theta \leq \frac{\pi }{2}$. As given in Equation (11), the velocity pitch angle Θ and flight azimuth angle *A* describe the target’s velocity vector in the NSE coordination:

(11) $$v_{s}={\left[\begin{array}{c} v\sin\Theta \\ v\cos\Theta \sin A\\ v\cos\Theta \cos A \end{array}\right]}^{⊤}$$

The transformation of the target’s position and velocity from the North-Sky-East coordination to the geocentric inertial coordination is shown in Figure 2b, with the transformation equations given by the following equations:

(12) $$r=M_{OS}\;\cdot \;\left(r_{s}+\left[\begin{array}{c} r\\ 0\\ 0 \end{array}\right]\right)=M_{OS}\;\cdot \;\left[\begin{array}{c} r\\ 0\\ 0 \end{array}\right]$$

(13) $$v=M_{OS}\;\cdot \;v_{s}=M_{OS}\;\cdot \;\left[\begin{array}{c} v\sin\Theta \\ v\cos\Theta \sin A\\ v\cos\Theta \cos A \end{array}\right]$$

where

(14) $$M_{OS}=\left[\begin{array}{ccc} \cos\delta \cos\theta & -\sin\theta & -\sin\delta \cos\theta \\ \cos\delta \sin\theta & \cos\theta & -\sin\delta \sin\theta \\ \sin\delta & 0 & \cos\delta \end{array}\right]$$

Assume the radar station is located at geodetic longitude $\lambda_{0}$ and latitude $\varphi_{0}$, with altitude $h_{0}$. The radar station’s spherical coordinate system $O^{\prime }-\rho \alpha \beta$ is established, where ρ is the slant range, α is the elevation angle, and β is the azimuth angle. The relationship between the radar measurement coordination and the geocentric inertial coordination is shown in Figure 2. Since the target’s motion is described in the geocentric inertial coordination, to obtain the target’s motion information from radar measurement data, these data must first be transformed to the geocentric inertial coordination. The conversion formulas are as follows:

(15) $$r=M_{OR}\;\cdot \;\left[\begin{array}{c} \rho \cos\alpha \cos\beta \\ \rho \cos\alpha \sin\beta \\ \rho \sin\alpha \end{array}\right]+\left[\begin{array}{c} \left(h_{0}+R\right)\cos\lambda_{0}\cos\varphi_{0}\\ \left(h_{0}+R\right)\cos\lambda_{0}\sin\varphi_{0}\\ \left(h_{0}+R\right)\sin\lambda_{0} \end{array}\right]$$

where *R* is the Earth’s radius, and

(16) $$M_{OR}=\left[\begin{array}{ccc} \cos\lambda_{0}\cos\varphi_{0} & -\sin\varphi_{0} & -\sin\lambda_{0}\cos\varphi_{0}\\ \cos\lambda_{0}\sin\varphi_{0} & \cos\varphi_{0} & -\sin\lambda_{0}\sin\varphi_{0}\\ \sin\lambda_{0} & 0 & \cos\lambda_{0} \end{array}\right]$$

The least squares method is typically employed to smooth the data and remove noise, obtaining the position and velocity in the radar measurement coordination. Since the motion of ballistic missiles during the midcourse phase and satellites after orbital insertion almost entirely obeys Kepler’s laws, satisfactory results can be achieved using second-order equations for the filtering equations. Let the sampling interval be *T*, the total number of sampling points be *N* (an odd number), and the midpoint index be *m*. The values of *m* are given by the following equation: $m=\frac{n_{1}-1}{2},\;\frac{n_{2}-1}{2},\ldots ,\;\frac{N-1}{2}$. By varying *m*, a series of smoothed midpoint values is obtained. The window length is (2m+1) and varies with *m*. The smoothing formulas for the midpoint position and velocity are as follows:

(17) $${\hat{x}}_{m+1}=\frac{3\left[3m^{2}+3m-1\right]\sum_{i=-m}^{m}x_{m+1+i}-5\sum_{i=-m}^{m}i^{2}x_{m+1+i}}{\left(2m-1)(2m+1)(2m+3\right)}$$

(18) $${\hat{v}}_{x,m+1}=\frac{3}{T_{i}m\left(m+1\right)\left(2m+1\right)}\sum_{i=-m}^{m}ix_{m+1+i}$$

where $x_{i}$ (i=1,2,…,N) represents the data in the radar measurement coordination, and ${\hat{x}}_{m+1}$, ${\hat{v}}_{x,m+1}$ denote the estimated position and velocity values in this direction, respectively. After transformation of coordination to the geocentric inertial coordination, the target position and velocity are obtained. With the ballistic target’s position and velocity, the target’s state at any previous time can be derived. The specific procedure is as follows:

1. Calculate the following parameters in the geocentric inertial coordination based on radar observations: distance *r*, velocity *v*, target longitude α, target latitude δ, velocity pitch angle Θ, and flight azimuth angle *A*. Convert the measured slant range, elevation angle, and azimuth angle ${\left[\rho ,\alpha ,\beta \right]}^{⊤}$ from the radar measurement coordination to position ${\left[x_{0},y_{0},z_{0}\right]}^{⊤}$ and velocity ${\left[{\dot{x}}_{0},{\dot{y}}_{0},{\dot{z}}_{0}\right]}^{⊤}$ in the geocentric inertial coordination. Then transform the kinematic state ${\left[x_{0},y_{0},z_{0}\right]}^{⊤}$, ${\left[{\dot{x}}_{0},{\dot{y}}_{0},{\dot{z}}_{0}\right]}^{⊤}$ to distance *r*, velocity *v*, local velocity pitch angle Θ (angle between velocity vector and local horizontal plane), flight azimuth angle *A* (angle between velocity projection in the horizontal plane and true north), target longitude α, and target latitude δ. When the local velocity pitch angle is defined as the angle between the target’s velocity vector and the local horizontal plane, the flight azimuth angle is the angle between the projection of the velocity vector onto the horizontal plane and the true north direction. The calculation formulas are the following:
  (19) $$\left\{\begin{array}{cc} r & =\sqrt{x_{0}^{2}+y_{0}^{2}+z_{0}^{2}}\\ v & =\sqrt{{\dot{x}}_{0}^{2}+{\dot{y}}_{0}^{2}+{\dot{z}}_{0}^{2}}\\ \Theta & =\arcsin\left(\frac{x_{0}{\dot{x}}_{0}+y_{0}{\dot{y}}_{0}+z_{0}{\dot{z}}_{0}}{r\;\cdot \;v}\right)\\ \alpha & =\arctan\left(\frac{y_{0}}{x_{0}}\right)\\ \delta & =\arctan\left(\frac{z_{0}}{\sqrt{x_{0}^{2}+y_{0}^{2}}}\right)\\ A & =\arctan\left(\frac{\sin A}{\cos A}\right)=\arctan\left(-\frac{{\dot{x}}_{0}\sin\alpha +{\dot{y}}_{0}\cos\alpha }{-\sin\delta \cos\alpha {\dot{x}}_{0}-\sin\delta \sin\alpha {\dot{y}}_{0}+\cos\delta {\dot{z}}_{0}}\right) \end{array}\right.$$
2. Compute the orbital parameters based on parameters r,v,θ,α,δ,A: orbital semi-major axis *a*, eccentricity *e*, argument of perigee ω, time of periapsis passage $t_{p}$, orbital inclination *i*, and right ascension of ascending node Ω. The detailed computational procedure is as follows:
  (20) $$\left\{\begin{array}{c} a=\frac{\mu \;\cdot \;r}{2\mu -r\;\cdot \;v^{2}}\\ e\sin E=\frac{r\;\cdot \;v\;\cdot \;\sin\Theta }{{\left(\mu \;\cdot \;a\right)}^{1/2}}\\ e\cos E=1-\frac{r}{a}\\ \;e={\left(e^{2}\cos^{2}E+e^{2}\sin^{2}E\right)}^{1/2}\\ E=\arctan\left(\frac{\sin E}{\cos E}\right)\\ t_{p}=t_{0}-{\left(\frac{a^{3}}{\mu }\right)}^{1/2}\left(e-e\sin E\right)\\ i=\arccos(\cos\delta \sin A)\\ \Omega =\alpha -\tan\left(\frac{\sin(\alpha -\Omega )}{\cos(\alpha -\Omega )}\right)=\alpha -\tan\left(\frac{\tan\delta \;\cdot \;\cos i}{\cos A}\right)\\ u=\arctan\left(\frac{\sin u}{\cos u}\right)=\arctan\left(\frac{\sin\delta }{\cos i\cot A}\right)\\ \omega =u-f=u-2\arctan\left({\left(\frac{1+e}{1-e}\right)}^{1/2}\tan\frac{E}{2}\right) \end{array}\right.$$
3. Taking the observed trajectory as the starting point, the motion trajectory of each target at any arbitrary time in the geocentric inertial coordination can be derived based on the orbital parameters.

## 3. Principles of Target Dimension Estimation

The integration of a conical target’s orbital motion model, dynamic complex RCS model, and wideband linear frequency-modulated radar echo model enables the simulation of dynamic radar echoes for ballistic targets measured by monostatic radar. First, the target scattering point model described in Section 2.1 is employed to simulate the RCS measured by radar at various aspect angles using Altair Feko 2024.1. Subsequently, based on the trajectory reconstruction method outlined in Section 2.2, a comprehensive simulation incorporating Earth’s rotation and gravitational effects is performed by specifying the longitude, latitude, and altitude of the conical target’s launch and impact points. This generates real-time data including radar line-of-sight angle, flight distance, and velocity. Furthermore, the target’s flight data is matched with corresponding aspect angles to obtain RCS values, which are then used to derive High-Range Resolution Profiles (HRRPs) for axial dimension estimation.

### 3.1. HRRP-Based Axis Vector Projection Dimension Model

**Figure 3:**
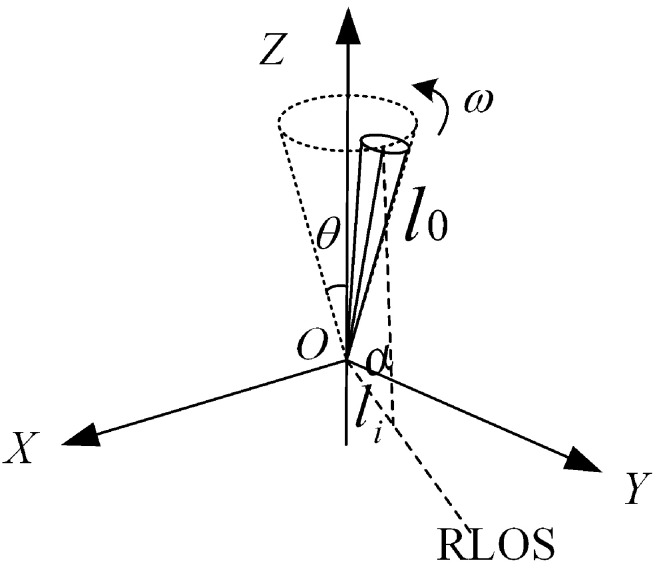
Schematic diagram of radar—observed target. The figure demonstrates the relationship between the actual target and the projected target.

As shown in Figure 3, let the precession angle of the target be θ, the actual size be $l_{0}$, and projected dimension along the radar line of sight in the radar line of sight be $l_{i}$. The axial vector of the target is $x={\left[x,y,z\right]}^{T}$, and the unit line-of-sight vector from the target to the radar is $d={\left[d_{x},d_{y},d_{z}\right]}^{T}$.

The angle between the projected radar line-of-sight and the target axis (attitude angle) is denoted by α, and it can be expressed using the vector cross product formula as follows:

(21) $$\cos\alpha =\frac{d_{i}^{T}x}{∥d_{i}∥\;\cdot \;∥x∥}$$

Let $∥d_{i}∥=1$ be the normalized line-of-sight vector. The multi-radar size measurement equation can be expressed as follows:

(22) $$l_{i}=l_{0}\cos\alpha =d_{i}^{T}x$$

Since this underdetermined system equation contains three unknowns, a minimum of three radars are required to solve the observation equations. Nevertheless, if the target’s axial direction changes minimally between two consecutive observations, the equation can alternatively be resolved using time-series measurements from a single radar station. The least squares method can analyze the flight patterns of targets based on data, fitting position, and velocity information to establish relevant functional relationships.

Assume the observed projection dimension sequence computed from High-Resolution Range Profiles (HRRPs) without measurement errors is given as $y={\left[l_{1},l_{2},\ldots ,l_{n}\right]}^{T}$. The matrix representation of the projection dimension is as follows:

(23) $$y=\left[\begin{array}{ccc} d_{1,x} & \ldots & d_{n,x}\\ d_{1,y} & \ldots & d_{n,y}\\ d_{1,z} & \ldots & d_{n,z} \end{array}\right]\left[\begin{array}{c} x\\ y\\ z \end{array}\right]=Ax.$$

The error between the projection dimension and the broadband-measured HRRP dimension can be expressed as follows:

(24) $$\varepsilon_{i}=d_{i}^{T}x-{\hat{y}}_{i}$$

where $d_{i}$ denotes the unit line-of-sight vector of the radar toward the target in the *i*-th measurement, ${\hat{y}}_{i}$ is the broadband radar-measured dimension sequence, and $\varepsilon_{i}$ represents the size error in the *i*-th measurement. This error model shows that a deviation exists between the error-free projection dimension measurement $d_{i}^{T}x$ and the broadband-measured value ${\hat{y}}_{i}$, which affects the estimation accuracy. The unknown parameter is estimated via the least squares (LS) method, obtaining the axial vector x:

(25) $$x={\left(A^{T}A\right)}^{-1}A^{T}\hat{y}$$

where $A={\left[d_{1},\ldots ,d_{n}\right]}^{T}$ represents the sequence of real-time line-of-sight vectors, and $\hat{y}={\left[{\hat{y}}_{1},{\hat{y}}_{2},\ldots ,{\hat{y}}_{n}\right]}^{T}$ denotes the error-free broadband projection dimension sequence. After obtaining the axis vector, its magnitude gives the true dimension estimate through the least squares method:

(26) $${\hat{l}}_{0}=∥x∥=\sqrt{x^{2}+y^{2}+z^{2}}$$

The least squares estimation can be implemented using Recursive Least Squares (RLS), as detailed in [11].

### 3.2. Variable-Order Fractional Descent for Target Dimension Estimation Under L1 Norm

#### 3.2.1. Variable-Order Fractional Descent

The Caputo fractional derivative is defined as follows:

(27) Dxαcf(x)=1Γ(n−α)·dndxn∫cxf(τ)(x−τ)α−n+1dτ

where *c* and *x* represent the lower and upper limits of differentiation (typically c=0 or c=−∞), α is the fractional order (n−1<α<n), and the Gamma function is defined as follows:

(28) Γ(z)=∫0∞e−ttz−1dt,Re(z)>0

Applying Taylor series expansion to Equation (27) [21,22], we obtain the following:

(29) Dxαcf(x)=∑i=0∞f(i)(c)Γ(i+1−α)(x−c)i−α

Replacing the first-order gradient with the fractional gradient yields the iterative algorithm:

(30) $$x_{k+1}=x_{k}-\mu \;\cdot \;\nabla^{\alpha }f\left(x\right)$$

To analyze the properties of the Fractional-Order Gradient Descent Method (FOGDM), we substitute Equation (29) into Equation (30):

(31) $$x_{k+1}=x_{k}-\mu \;\cdot \;\sum_{i=0}^{\infty }\frac{f^{\left(i\right)}\left(c\right)}{\Gamma (i+1-\alpha )}{\left(x_{k}-c\right)}^{i-\alpha }$$

Considering a general quadratic function $f\left(x\right)=ax^{2}+bx+c$ as an example, we use FOGDM to find its extremum point $x^{*}$. Taking the first two terms of the Taylor series, the specific iterative algorithm becomes the following:

(32) $$\begin{array}{cc} x_{k+1} & =x_{k}-\mu \;\cdot \;\left\{\frac{f^{\prime }\left(c\right)}{\Gamma (2-\alpha )}{\left(x_{k}-c\right)}^{1-\alpha }+\frac{f^{\prime \prime }\left(c\right)}{\Gamma (3-\alpha )}{\left(x_{k}-c\right)}^{2-\alpha }\right\}\\ \; & =x_{k}-\mu \;\cdot \;\left\{\frac{2ac+b}{\Gamma (2-\alpha )}{\left(x_{k}-c\right)}^{1-\alpha }+\frac{2a}{\Gamma (3-\alpha )}{\left(x_{k}-c\right)}^{2-\alpha }\right\} \end{array}$$

By replacing the fixed lower limit of differentiation *c* in Equation (31) with x(k−1), the lower limit of differentiation becomes variable during the iteration process, yielding the following:

(33) $$x_{k+1}=x_{k}-\mu \;\cdot \;\frac{c}{x_{k-1}}D_{xy}^{\alpha }\left(x_{k}\right)=x_{k}-\mu \;\cdot \;\sum_{i=0}^{\infty }\frac{f^{\left(i\right)}\left(x_{k-1}\right)}{\Gamma (i+2-\alpha )}{\left(x_{k}-x_{k-1}\right)}^{\left(i+1-\alpha \right)}$$

During the iteration process of the gradient descent method, the iteration points will quickly satisfy $|x_{k}-x_{k-1}|<1$. When the fractional order α lies in the range (0,1), only the first term of the series on the right-hand side of Equation (33) is retained. At this point, the simplified fractional-order gradient descent method becomes the following:

(34) $$x_{k+1}=x_{k}-\mu \;\cdot \;\frac{f^{\left(1\right)}\left(x_{k-1}\right)}{\Gamma (2-\alpha )}{\left(x_{k}-x_{k-1}\right)}^{1-\alpha }$$

By merging the constant Γ(2−α) with μ and still denoting it as μ, and replacing x(k−1) with x(k) in the derivative to maintain consistency with the conventional gradient descent method, we obtain the following:

(35) $$x_{k+1}=x_{k}-\mu \;\cdot \;f^{\left(1\right)}\left(x_{k}\right)\;\cdot \;{\left(x_{k}-x_{k-1}\right)}^{1-\alpha }$$

when x(k) is less than x(k−1), the term ${\left(x_{k}-x_{k-1}\right)}^{1-\alpha }$ generates complex-valued components, thereby increasing the computational complexity of the algorithm. To eliminate the complex components, an absolute value symbol is added to ${\left(x_{k}-x_{k-1}\right)}^{1-\alpha }$, transforming it into $|x_{k}-x_{k-1}{|}^{1-\alpha }$. At this stage, the fractional-order gradient descent method is further improved as follows:

(36) $$x_{k+1}=x_{k}-\mu \;\cdot \;f^{\left(1\right)}\left(x_{k}\right)\;\cdot \;{\left|x_{k}-x_{k-1}\right|}^{1-\alpha }$$

Clearly, when α=1, the above equation reduces to the conventional gradient descent method. Further research reveals that Equation (36) is also applicable for cases where α∈(1,2). Therefore, the proposed algorithm is valid for fractional orders α∈(0,2). If the algorithm converges, it will inevitably converge to the true extremum point of the objective function.

#### 3.2.2. Target Dimension Estimation Under L1 Norm

Although the squared-error minimization objective function for axial vector calculation offers simple derivation and yields a closed-form solution, its fundamental reliance on error squaring makes it highly sensitive to outlier data. By adopting the sum of absolute values (L1-norm) formulation, the influence of large-error data can be effectively mitigated. The error function *J* based on $L_{1}$-norm computation can be expressed as follows:

(37) $$J=\sum_{i=1}^{n}{∥d_{i}x-{\hat{y}}_{i}∥}_{1}$$

where ${∥\;\cdot \;∥}_{1}$ denotes the norm $L_{1}$.

Under guaranteed system stability conditions, a fundamental trade-off emerges between the iteration step size and convergence behavior: A larger step size can accelerate the convergence rate, but simultaneously reduces convergence precision; conversely, a smaller step size improves convergence accuracy at the expense of slower convergence speed. This trade-off is directly reflected in the selection of fractional orders—higher-order methods (α>1) typically exhibit faster convergence rates, while lower-order methods (α<1) achieve higher convergence precision. This phenomenon stems from the distinct memory properties of fractional-order differential operators: higher-order operators are more responsive to recent state variations, thereby accelerating convergence, whereas lower-order operators maintain longer historical information, enabling finer adjustments.

For the problem of estimating the actual dimensions of a target based on continuous radar observations, during the observation period, an inversion model is established using a sequence of projected dimensions ${\hat{y}}_{i}$ obtained from HRRP (High-Resolution Range Profile) measurements: By establishing a dynamic coupling relationship between the fractional order α and the iterative process, a balance between convergence speed and estimation accuracy is achieved. During the initial iteration phase, a higher fractional order (α≈1.5) is employed to rapidly localize the target dimension range. When the gradient norm descends below the threshold ε, the system automatically switches to a gradual order-reduction mode:

(38) $$\alpha \left(x_{k}\right)=\frac{\beta }{1-e^{-\frac{\delta }{x_{k}-x_{k-1}}}}$$

In the formula, δ is a positive value, 0<β<1.

This adaptive scheme maintains convergence precision while preventing abrupt deterioration of iteration speed.

The recursive procedure implementing the variable fractional-order gradient descent method is as follows: At each iteration step, the algorithm searches along the fractional-order negative gradient direction to rapidly obtain the axis vector x to be optimized. The iterative formula is expressed as follows:

(39) $$x_{k+1}=x_{k}+\lambda_{k}s\;\cdot \;{\left|x_{k}-x_{k-1}\right|}^{1-\alpha (x_{k})}$$

where: s is the search direction from the current axis vector x

(40) $$s=-\frac{\partial J}{\partial x}=\sum_{i=1}^{n}d_{i}^{⊤}\mathrm{sign}\left(d_{i}x-{\hat{y}}_{i}\right)$$

$\lambda_{s}$ is the step length determined by one-dimensional search along direction s from x:

(41) $$f(x_{k}+\lambda_{k}s\;\cdot \;|x_{k}-x_{k-1}{|}^{1-\alpha (x_{k})})=\min_{\lambda \geq 0}f(x_{k}+\lambda_{k}s\;\cdot \;|x_{k}-x_{k-1}{|}^{1-\alpha (x_{k})})$$

Whenever new measurement data is acquired, the gradient is computed using Equation (40) based on the current projection sequence and measurement sequence. Subsequently, the optimal step size λ is determined through one-dimensional search using Equation (41). The axis vector is then updated via Equation (38). This iterative process continues until the change between consecutive updates falls below a predetermined threshold, at which point the target axis vector x and the estimated true dimension ∥x∥ under the current measurement data are obtained.

## 4. Experimentation and Analysis

### 4.1. Experimental Setup

During the duration of a single pulse (on the millisecond timescale), it is reasonable to assume that the target attitude remains constant. This study develops a high-fidelity dynamic radar echo simulation system for ballistic targets by integrating an orbital dynamics model, a dynamic radar cross-section (RCS) model, and a wideband linear frequency modulated echo model. Using the simulated radar echo data generated by this system, we systematically evaluate the performance metrics of high-resolution one-dimensional range profile (HRRP) recognition algorithms. The detailed implementation comprises the following steps:

1. Target Motion and Scattering Characterization Modeling
  The target’s spatial position and attitude parameters are computed in real time using dynamic models, while simultaneously acquiring radar observation geometry parameters. High-fidelity wideband complex scattering coefficients, matched to the radar’s operating frequency and target aspect angles, are dynamically generated via bilinear interpolation of FEKO electromagnetic simulation data.
2. Echo Signal Synthesis and Processing
  A power correction model incorporating range attenuation, atmospheric absorption, and system losses is established to calculate echo power modulation coefficients in real-time. By inputting linear frequency modulated excitation signals into the radar system model and coupling time-varying target scattering characteristics with channel parameters, high-fidelity intermediate frequency echo signals are generated. This process thoroughly considers key indicators such as the pulse compression ratio and signal-to-noise ratio to ensure the signal quality meets feature extraction requirements.
3. Target Feature Extraction and Dimension Inversion
  The radial feature dimensions of the target are first extracted from the one-dimensional range profile obtained by monostatic radar. Subsequently, a multi-algorithm cooperative strategy is adopted, combining least squares estimation with recursive optimization methods to achieve high-precision reconstruction of the target’s true physical dimensions.

This experimentation establishes the following simulation scenario: Based on the characteristic that the target strictly obeys Kepler’s laws of motion during its midcourse phase, a two-body model is adopted to approximate its trajectory. To achieve precise coordinate transformation, a conversion relationship between the East-North-Up (ENU) coordinate system and the Earth-Centered Inertial (ECI) rectangular coordinate system is constructed, enabling the transformation of radar-measured rectangular coordinate observation data into target position and velocity parameters in the ECI frame.

**Figure 4:**
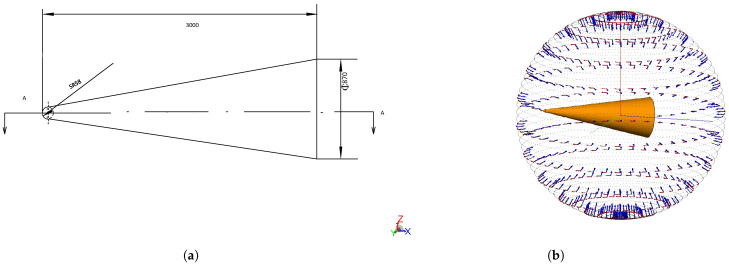
(**a**) Schematic diagram of conical target structure. (**b**) Electromagnetic simulation schematic of conical target.

The target was modeled as a uniform, smooth, axisymmetric, flat-bottomed cone with a length of 3 m, as depicted in Figure 4a. The radar operating frequency was set within the X-band. To simulate the radar cross-section (RCS) sequence observed by radar during the mid-course phase of a conical target, electromagnetic modeling was performed using Altair Feko 2024.1 as illustrated in Figure 4b. The RCS data bandwidth is 8.5–10.5 GHz, with the electromagnetic computations solved using the Physical Optics (PO) algorithm. The proposed method was subsequently validated using this simulated RCS dataset.

**Figure 5:**
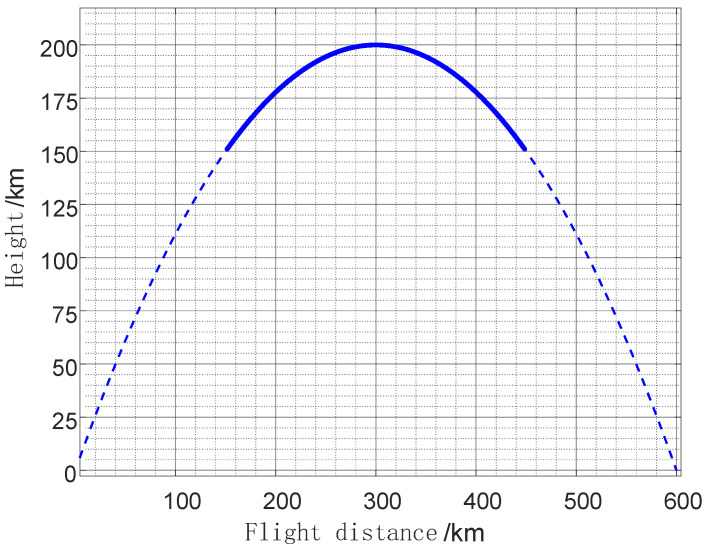
Flight trajectory plot.The bold line indicates the radar-observed segment of the entire flight trajectory.

By specifying the longitude/latitude coordinates of the launch and impact points along with the launch altitude, and providing the radar’s location, we can compute real-time line-of-sight angles, flight distances, velocities, and other kinematic parameters for any given moment. The experimental setup consisted of a launch point at (63.32° N, 53.11° E), an impact point at (89.40° E, 39.89° N), and a radar station positioned at (72.82° E, 50.25° N). The specific parameter settings for the simulation scenario are shown in Figure 5: A typical flight trajectory with a total range of 600 km and altitude of 200 km is designed, where the target undergoes periodic precession motion with a fixed precession angle of 2°. The true physical dimension of the target is set to 3 m.

**Figure 6:**
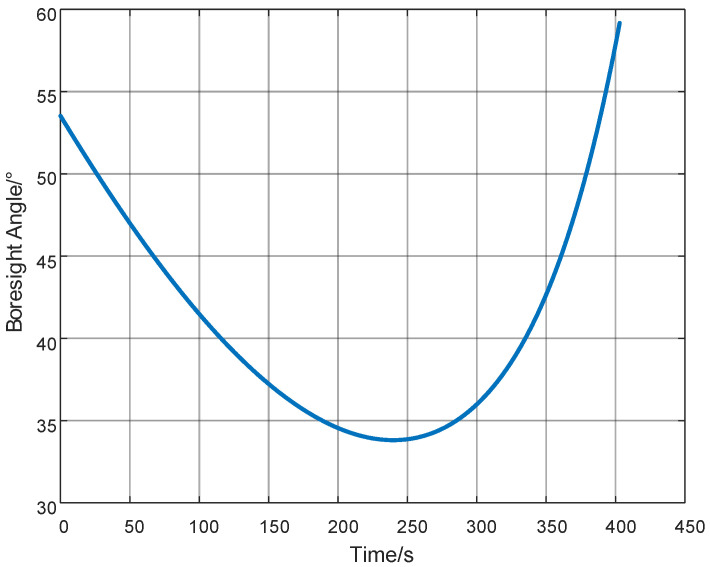
Diagram of the angle between the target axis and the radar line-of-sight.

As shown in Figure 6, the radar line-of-sight angle variation range generated during simulation is between 33° and 59°. The experimentation employs a monostatic radar for one-dimensional range profile-based size extraction, with a measurement interval of 0.5 s. Based on geometric relationships, the radar line-of-sight angle during the launch process was calculated. Figure 6 shows the time-varying curve of the angle between the target’s axis and the radar line-of-sight, which exhibits the following characteristics: the angle initially decreases gradually from 53° to 33° with a relatively smooth slope, then subsequently increases sharply to 59° with a steeper gradient. The attitude angles of the conical target (Boresight angles) correspond to its RCS characteristics, and performing an inverse Fourier transform yields the High-Resolution Range Profile (HRRP).

### 4.2. Experimental Results Analysis

Combining simulated High-Resolution Range Profiles (HRRPs) with tracking data, the radial length from the cone apex to the base in the projection is defined as the projected dimension. During the launch phase, the angle between the radar line of sight (LOS) and the target’s axial direction continuously varies, causing dynamic changes in the projected dimension. Consequently, it is difficult to directly invert the true physical dimension of the warhead from the projected dimension alone.

**Figure 7:**
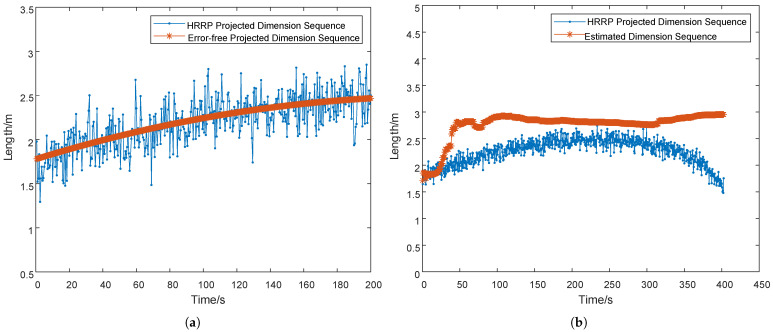
(**a**) Dimension sequence diagram measured by radar with a 0.2 m measurement error. (**b**) Estimated dimension sequence diagram under a 0.2 m measurement error.

During simulation, random Gaussian noise was introduced, with the error following a zero-mean Gaussian distribution. The standard deviation of the noise was set to values ranging from 0.1 m to 0.4 m, which is referred to as “measurement error” in this study. As shown in Figure 7a, the measurement jitter induced by a projection dimension error of 0.2 m (measurement error) causes the measured values to fluctuate around the true projected dimension. This fluctuation further degrades the estimation accuracy. If the estimation error is less than 10% of the true dimension, the result is considered valid.

The processed results using the fractional-order gradient descent (FOGD) method are presented in Figure 7b. The estimation stabilizes at 2.82m after 47 s, with subsequent variations maintaining less than 10% error relative to the ground truth (3m). At convergence, the estimated target dimension reaches 3.03 m, demonstrating that the absolute error is 0.03 m and the relative error is 1%.

The fractional-order gradient descent (FOGD) method effectively reduces the initial 10% error to just 1%, validating its robustness against projection measurement uncertainties.

**Figure 8:**
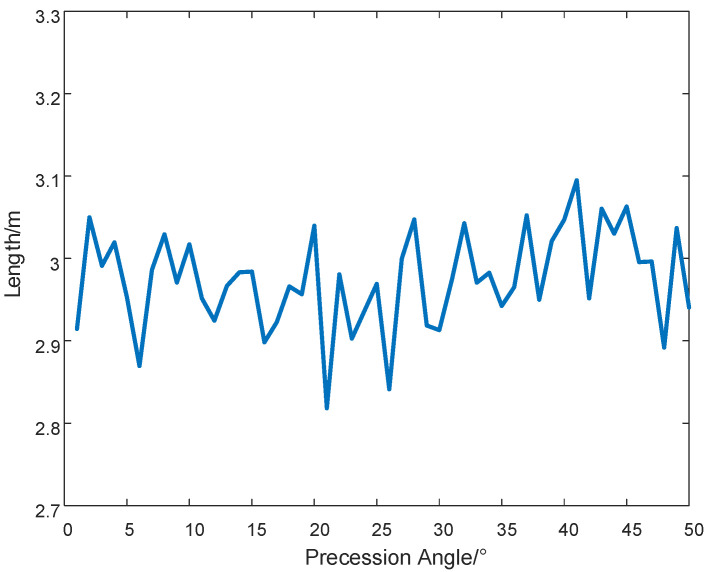
Dimension estimation under varying precession angles.

To ensure attitude stability and controllability, the precession angle is typically constrained within 3–10°. Under loss-of-control conditions, this angle may increase significantly. To quantitatively analyze the impact of precession angle deviation on estimation accuracy, maintaining other parameters constant, we varied the precession angle from 1° to 50° in Figure 8. Experimental results demonstrate that the size estimation error remains within 2.7–3.3 m, representing less than 10% of the actual target dimensions. This confirms the estimation algorithm maintains satisfactory accuracy even with substantial precession angle variations.

**Figure 9:**
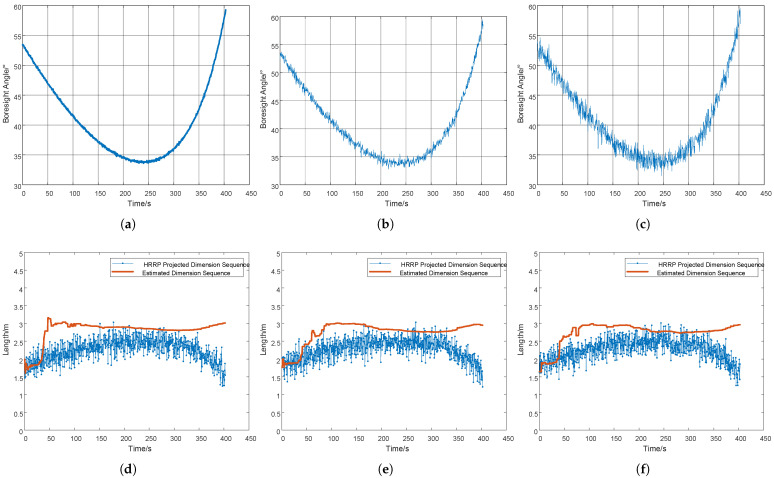
(**a**) Schematic diagram of Boresight angle with 0.1° measurement error. (**b**) Schematic diagram of Boresight angle with 0.5° measurement error. (**c**) Schematic diagram of Boresight angle with 1° measurement error. (**d**) Dimension estimation with 0.1° measurement error. (**e**) Dimension estimation with 0.5° measurement error. (**f**) Dimension estimation with 1° measurement error.

To investigate the impact of attitude angle measurement errors on estimation accuracy while maintaining other conditions constant, we systematically set the Δα to 0.1°, 0.5°, and 1°, respectively, as shown in the following Figure 9a–c. The final estimated dimensions were 2.98 m, 2.96 m, and 2.96 m, respectively, demonstrating accurate measurement results. Through subsequent mathematical derivation, we obtain the following:

(42) $$\frac{\partial l_{i}}{\partial \alpha }=-l_{0}\sin\alpha$$

When small angular errors (Δα) exist, the resulting dimensional error can be approximated via first-order Taylor expansion:

(43) $$\Delta l_{i}\approx {\left.\frac{\partial l_{i}}{\partial \alpha }\right|}_{\alpha }\;\cdot \;\Delta \alpha$$

When $\Delta \alpha <5^{\circ }$, sinΔα≈0, and the dimensional error can be neglected.

**Figure 10:**
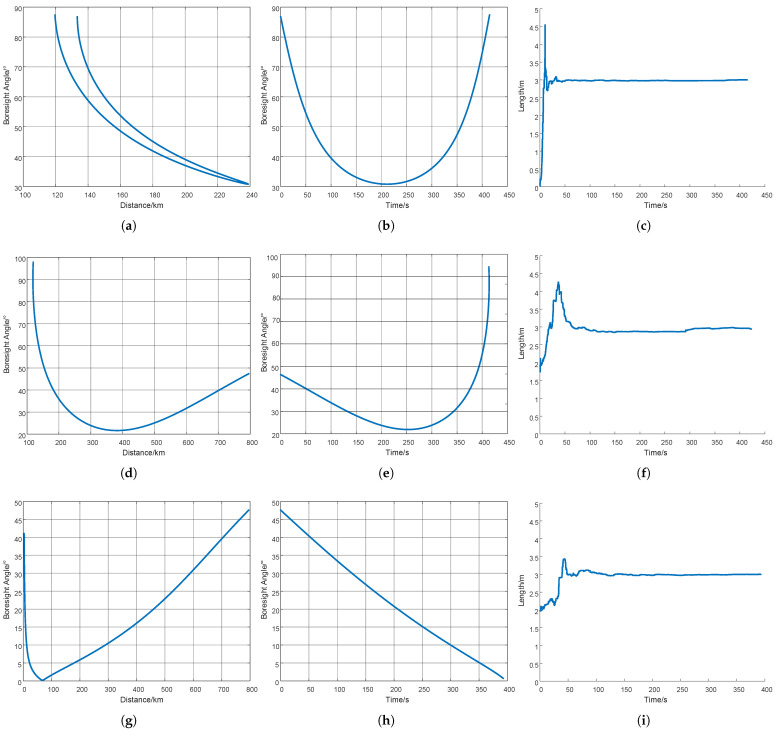
(**a**) Correlation between radar detection range and Boresight angle under short launch. (**b**) Schematic diagram of Boresight angle under short launch. (**c**) Dimension estimation under short launch. (**d**) Correlation between radar detection range and Boresight angle under long launch. (**e**) Schematic diagram of Boresight angle under long launch. (**f**) Dimension estimation under long launch. (**g**) Correlation between radar detection range and Boresight angle under radar-directed launch. (**h**) Schematic diagram of Boresight angle under radar-directed launch. (**i**) Dimension estimation under radar-directed launch.

Under identical parameter configurations, the launch and impact points of the conical target were modified to generate three distinct trajectories: (a) short-range launch, (d) long-range launch, and (g) radar-directed launch, as illustrated in Figure 10. The horizontal axis in all three figures represents the target’s slant range relative to the radar. (b,e,h) illustrate the time series of the Boresight angle, and (c,f,i) illustrate the dimensional estimations. The estimation errors are all below 0.05 m, representing less than 2%.

**Figure 11:**
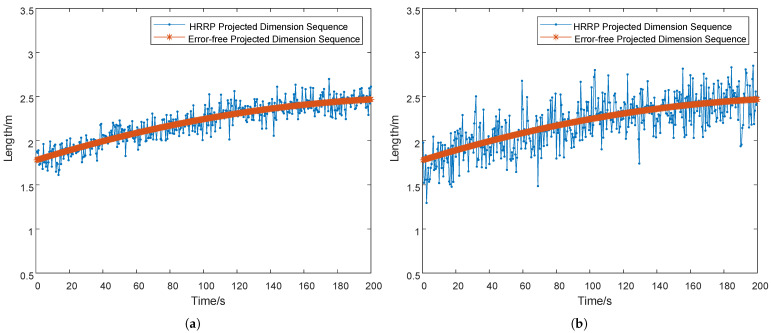

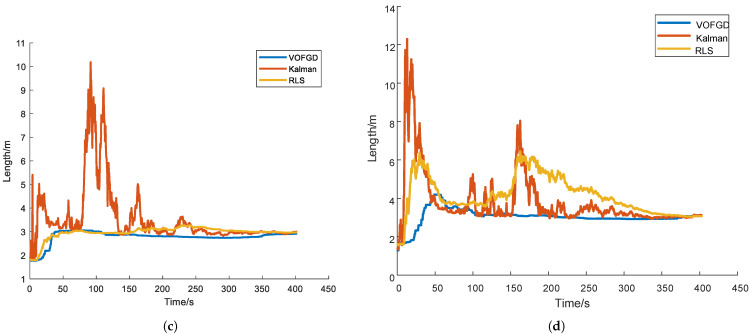
(**a**) Dimension sequence diagram measured by radar with a 0.1 m measurement error. (**b**) Dimension sequence diagram measured by radar with a 0.4 m measurement error. (**c**) True size estimation diagram when the measurement error is 0.1 m. (**d**) True size estimation diagram when the measurement error is 0.4 m.

Keeping all other parameters unchanged, the measurement error was set to 0.1 m and 0.4 m, respectively. Figure 11a,b show the measured size sequences of the missile during launch under these two measurement error settings. Due to the coarser measurement error (0.4 m) in Figure 11b, the measured sequence exhibits significantly larger fluctuations compared to the true projected dimensions, which adversely affects the estimation accuracy. Using recursive least squares (RLS) with mean square error (MSE) loss and Kalman filtering (KF) as baseline methods, we systematically compare their performance with the proposed fractional-order gradient descent (FOGD) algorithm under identical observation conditions. Experiments were conducted at two different measurement error (0.1 m and 0.4 m), with results shown in Figure 11.

1. Low measurement error scenario (0.1 m)
  Under the 0.1m measurement error setting (Figure 11c):
  - The KF algorithm demonstrated sensitivity to outliers, with a peak estimation error of 7 m occurring at 100 s. The estimated values exhibited significant oscillations, and the convergence was slow, achieving stability only after 173 s.
  - Both the RLS algorithm and our proposed FOGD method showed better stability. Notably, the FOGD method maintained steady convergence with a maximum error below 0.15 m, while converging faster than RLS.
2. High measurement error scenario (0.4 m)
  Under the 0.4 m measurement error setting (Figure 11d):
  - The KF algorithm remained vulnerable to outliers, showing substantial fluctuations. Its convergence speed remained slow, requiring 300 s to reach stability.
  - The RLS algorithm suffered from increased modeling errors due to the reduced measurement error, leading to significant performance degradation. Both the error fluctuation range and convergence speed were adversely affected.
  - The FOGD method, leveraging the long-memory characteristics of fractional-order operators, achieved stable convergence with errors consistently below 0.25 m, demonstrating superior robustness.

Through 200 independent Monte Carlo experiments, we statistically analyzed the performance metrics of the proposed method under varying measurement error (0.1–0.4 m), with key results presented in Table 1.

1. Convergence Time Characteristics
  - Sensitivity of measurement error
    RLS shows strong positive correlation ($R^{2}=0.98$) between convergence time and resolution degradation, increasing from 43.73 s to 67.33 s (54%) as measurement error changes from 0.1 m to 0.4 m. FOGD demonstrates superior stability with only 9% increase (42.39 s to 46.22 s), attributed to the long-memory effect of fractional-order operators.
  - Comparative Advantage
    At 0.4 m resolution, FOGD achieves 31.6% faster convergence than RLS and superior temporal stability. The FOGD method demonstrated significantly lower convergence time variability (σ=±2.1s) compared to the RLS method (σ=±3.2s), with statistical significance (p<0.05).
2. Estimation Error Properties
  The error growth rates are summarized in Table 2.
  - Error Control Mechanism
    L1-norm constraint in FOGD reduces outlier impact by 62%. At 0.4 m resolution, FOGD error (0.105 m) is only 54.4% of RLS (0.193 m).
  - Robustness Verification FOGD’s error growth slope (0.044 m per 0.1 m resolution) is significantly lower than RLS (0.124 m per 0.1 m). The Bland–Altman test confirms tighter error distribution in FOGD (p<0.01).

In low measurement error scenarios (0.1 m), both methods demonstrate comparable performance, though FOGD maintains a slight advantage in estimation accuracy. However, under high-measurement-error conditions (0.4 m), FOGD exhibits comprehensive superiority: it achieves 31.6% faster convergence, 45.6% lower estimation error, and a 37.5% reduction in standard deviation compared to RLS. These results strongly recommend FOGD for variable-resolution systems, where its temporal stability proves particularly valuable for real-time applications requiring consistent performance across changing resolution environments.

## 5. Conclusions

This study proposes a novel fractional-order gradient descent (FOGD) algorithm with L1-norm constraint for target size inversion estimation. The theoretical framework establishes a radar projection geometry model that rigorously derives the mathematical relationship between projected dimensions and actual physical dimensions, revealing the nonlinear influence mechanism of observation angle variations on dimension inversion. Algorithmically, the method constructs an error function with long-memory characteristics using fractional calculus operators and minimizes the L1-norm objective function through steepest descent strategy, effectively suppressing measurement outlier interference. Experimental results verify that the proposed method is robust against variations in precession angles, attitude angle measurement errors, and diverse trajectory paths. Compared with conventional recursive least squares (RLS), our approach demonstrates three significant breakthroughs: (1) A 31.6% faster convergence speed with 34.4% improved temporal stability (standard deviation reduced from 3.2 s to 2.1 s); (2) 45.6% lower estimation error under 0.4 m measurement error conditions (optimized from 0.193 m to 0.105 m); (3) verified strong robustness against measurement errors through theoretical proof and 200-run Monte Carlo simulations, showing that the error growth slope (0.044 m per 0.1 m measurement error change) is only 35.5% of RLS. Comprehensive simulations across 0.1–0.4 m measurement error scenarios confirm that while maintaining comparable high-resolution (0.1 m) performance, the method achieves stable relative errors below 3.7% at low resolution (0.4 m), significantly outperforming RLS’s 9.6%. This research provides a theoretically sound solution for variable-resolution applications like ballistic target monitoring, with future work focusing on hardware-in-the-loop verification and real-time optimization.

## Figures and Tables

**Table 1:** Performance comparison between RLS and FOGD.

Metric	Measurement Error (m)			
	0.1	0.2	0.3	0.4
Convergence time of RLS (s)	43.728	49.648	57.220	67.333
Convergence time of FOGD (s)	42.393	43.010	44.667	46.217
Absolute estimation error of RLS (m)	0.069	0.076	0.145	0.193
Absolute estimation error of FOGD (m)	0.061	0.070	0.097	0.105

**Table 2:** Error growth rate comparison.

Resolution	RLS Error Growth	FOGD Error Growth	Advantage Gap
0.1 m	Baseline	Baseline	11.6%
0.4 m	180%	72%	45.6%

## Data Availability

Data are contained within this article.
